# Chemical Composition, Antioxidant, Genotoxique and Antigenotoxic Potentials of *Phlomis Bovei *De Noé Aerial Parts.

**DOI:** 10.22037/ijpr.2019.15197.12938

**Published:** 2020

**Authors:** Nabila Zaabat, Anne-Emmanuelle Hay, Serge Michalet, Inès Skandrani, Leila Chekir-Ghedira, Marie-Geneviève Dijoux-Franca, Salah Akkal

**Affiliations:** a UMR 5557 CNRS-UCBL – Ecologie Microbienne, Université Lyon 1, Villeurbanne, France.; b Département de Chimie, Laboratoire de Recherche en Phytochimie et Analyse Physicochimique, Université Mentouri, Constantine, Algeria.; c Laboratoire de Biologie Moléculaire et Cellulaire, Faculté de Médecine Dentaire de Monastir, Tunisia.

**Keywords:** Phlomis bovei De Noe, Chemical constituents, Antioxidant, Genotoxic effect, Antigenotoxic activity

## Abstract

In the present work, chemical investigation of the aerial parts of *Phlomis* *bovei* de Noé an endemic species from Algeria, led to the isolation and identification of seven known compounds including five flavones glycosides: Chrysoeriol 7-*O*-(3’’-(*E* et *Z*)-*p*-coumaroyl)-β-glucoside **(1)**, terniflorin (apigenin-7-*O*-(6’’-*E-p*-coumaroyl)glucoside) **(3)**, apigenin-7-*O*-(6’’-(5’’’-methoxy-coumaryl) glucoside **(4)**, apigenin 7-*O*-(3″-*p*-coumaryl)*glucoside* **(5)**, hispidulin-7-*O*-glucuronide **(6)** and two cinnamic acid derivatives: *p*-coumaric acid methyl ester (*E* et *Z*) **(2)**, chlorogenic acid **(7)**. Compound **4** is described for the first time in the species *bovei* de Noé, the genus *Phlomis* and the *Lamiaceae* family. Structures elucidation was performed by comprehensive 1D and 2D NMR analyses, mass spectrometry and by comparison with literature data. Some pure compounds and extracts have been evaluated for their antioxidant activities through different methods: DPPH and ABTS assays as well as CUPRAC assay. Genotoxic and antigenotoxic activities of pure compounds were also evaluated *in-vitro* on *Escherichia coli* PQ37 cells by the SOS Chromotest.

## Introduction

The genus *Phlomis* (Lamiaceae) includes about 100 species, which are widespread in Africa, Asia, and Europe. This genus is divided into two sections *Phlomis* and *Phlomoides*. The genus *Phlomis* is widely used in traditional medicine in many areas. In Algeria this genus is traditionally used to treat inflammation and rheumatism (1). In Turkey, most species in this genus are used as a tonic or stimulant (2). In China, 43 other species are the most popular herbs in China due to their aromatic and medicinal properties (3).

The biological investigation and the chemical studies of extracts of the genus *Phlomis* have shown that they possess numerous biological activities. For example, aqueous extract of aerial parts of *Phlomis* *crinita* and *Phlomis* *grandiflora* are reported for treating gastric ulcers (4, 5).

Iridoids, flavonoids, phenylpropanoids, phenylethanoids, lignans, neolignans, diterpe-noids, alkaloids, and essential oils are typical metabolites of the *Phlomis *genus (6).

*Phlomis* *bovei* De Noé subsp *bovei* known as Kayat El Adjarah or Tarseouan is one rare and endemic of the four species that are encountered in Algeria. This plant is used as a glue, and as a healing to treat burns lesions and skin infections and allergies (7). The main components of *Phlomis bovei*, previously reported were flavones and sterol (8). In this paper we described the isolation and identification of five more flavonoids of *Phlomis bovei*. Their structures were elucidated on the basis of nuclear magnetic resonance (NMR) analyses, including one-dimensional (1D) and two-dimensional (2D) NMR experiments, mass spectrometry and by comparison with literature data. 

Some pure compounds and extracts have been evaluated for their antioxidant activities through different methods: DPPH and ABTS assays as well as CUPRAC assay. Genotoxic and antigenotoxic activities of pure compounds were also evaluated *in-vitro* on *Escherichia coli* PQ37 cells by the SOS Chromotest.

## Experimental

*General experimental procedures*

NMR spectra were recorded on a Brucker DRX 500 Spectrometer (500 MHz for ^1^H and 125 MHz for ^13^C) in CDCl_3_ as solvent (internal reference, TMS). ESI-MS were recorded with a Thermo LCQ advantage, ion-trap spectrometer while HR-ESI/MS were recorded with Thermo Finnigan Mat 95 XL. UV spectra were recorded on a Shimadzu UV-1240 spectrophotometer. Vacuum liquid chromatography (VLC): silica gel 60 (40–63 μm) (Merck). Column chromatography (CC): Sephadex LH20; silica gel (60–200 μm) (Merck); Analytical and preparative TLC were carried out using: Merck silica gel Si 60 F254 (20 × 20 cm) or RP18 F254 (20 × 20 cm) or polyamide 11F254 (20 ×20 cm) aluminum sheets. For SPE, plastic syringes were filled with reverse phase, Merck LiChroprep RP-18 60 (40–63 μm),

*Plant material*

Aerial parts from *Phlomis bovei (P. bovei) *de Noé were collected in mai 2013 in Algeria. They were identified by Prof. Hocine Laouer (Department of Biology, Faculty of sciences, University Ferhat Abbas, Setif, Algeria). A voucher specimen is deposited in the herbarium of the Department of Nature and Life Sciences, University of Ferhat Abbas Setif, Algeria, under the code Phb-012-4-2013.

*Extraction and isolation*

The air dried powdered aerial parts of *P. bovei *de Noé (1.5 kg) were successively extracted with *n*-hexane, CH_2_Cl_2_, MeOH, and (MeOH-H_2_O) at room temperature for 48 h. Ninety-five gram of the MeOH extract was fractionated over silica gel–VLC eluting with CH_2_Cl_2_ followed by increasing concentrations of MeOH affording eight fractions A1–A8. Fractions A3 and A4 (41 g), eluted with CH_2_Cl_2_–MeOH (50:50–25:75), were combined and further applied to column chromatography over silica gel using CH_2_Cl_2_–EtOAc and EtOAc–MeOH, 6 fractions were collected (B1–B6). Compound **1** was obtained from B2 (284 mg) after successive RP18 SPE with a gradient H_2_O–MeOH, a Sephadex LH20 using MeOH and a preparative polyamid TLC using (toluene–MeOH–Methyl Ethyl Cetone) (3:1:1). Purification of (1,73 g) fraction B3 by RP18 MPLC with (H_2_O+0,01TFA) –(MeOH+0,01TFA) to yield six fractions (C1-C6). An SPE of C4 (112 mg) over RP-18 with H_2_O-MeOH (100:0 to 0:100) as eluent has been used, then a preparative silica gel TLC using CH_2_Cl_2_-MeOH (90:10) which have permitted to isolate compound **2**. Fraction C5 (52 mg) was subjected to flash chromatography on RP-18 using a gradient H_2_O- MeOH (60:40 to 0:100) to give compounds **4**, **5,** and **6**. B5 (600 mg) was purified on a Sephadex LH20 with MeOH:H_2_O (8-2) to yield compounds **6** and **7**.

*Genotoxicity assay*

The SOS Chromotest employs the error-prone DNA repair pathway of *Escherichia coli* PQ37, also known as the SOS response, a complex regulatory network that is induced by DNA-damaging substances (9). The test involves incubation of the bacteria with the sample under investigation and subsequent determination of b-galactosidase activity. The test was performed as recommended by Quillardet and Hofnung (10) by dividing the reaction mixtures into two series: one for β-galactosidase (β-gal) activity measurement (inducible) and the other for alkaline phosphatase (AP) (constitutive) to control the protein synthesis. The enzymatic activities were measured at 420 nm using a blank without bacteria. Positive control consisted of nitrofurantoin (NF). The induction factor (IF) was calculated as the ratio of Rc/R_0_, where Rc is equal to (β-gal activity/AP activity) determined for the test compound at concentration c and R_0_ is equal to (β-gal activity/AP activity) in the absence of test compound. The β-galactosidase and alkaline phosphatase activities were calculated according to the method recommended by Quillardet and Hofnung (11). The compounds are classified as non-genotoxic if the IF (Induction Factor) remains <1.5, as marginally genotoxic if the IF ranges between 1.5 and 2 and as genotoxic if the IF exceeds 2 (12). The data were analyzed for statistical significance using the Duncan test.

In a series of experiments preceding the SOS Chromotest assay, it was ascertained, by using the method of disc, that the different amounts of extracts and molecules added to the indicator bacteria does not influence their viability, and does not provoke a significant decrease of alkaline phosphatase activity, which is an indicator of the normal protein synthesis in *Escherichia coli* PQ37.

*Antigenotoxicity assay*

The antigenotoxicity of extracts and compounds against nitroxazide (10 µg/assay) damage was studied. The compounds were dissolved in dimethylsulfoxide (DMSO), three concentrations of each compound (2, 10 and 50 µg/assay) and of each extract (10, 50, and 250 µg/assay) were prepared and tested in triplicate. Percentages of antigenotoxicity (%AG) were determined according to the Equation: (AG%) = 100 – [(IF_1_ - IF_0_)/IF_2_ - IF_0_)] × 100.

Where IF_1_ is the IF in the presence of the test compound and the genotoxin, IF_2_ is the IF in the absence of the test compound and in the presence of the genotoxin and IF_0_ is the IF of the untreated cells. The data were collected with a mean ± standard deviation of three independent experiments, and analyzed for statistical significance using the Dunett test.

*DPPH free radical-scavenging activity*

Free radical scavenging capacity of the purified compounds was determined with the 1,1-diphenyl-2-picrylhydrazyl (DPPH) assay (13). The molecule DPPH is characterized as stable-free radical by virtue of the delocalization of the spare electron over the molecule; this delocalization gives rise to a deep violet color, characterized by an absorption band in methanol solution centered at 515 nm with a spectrophotometer. This assay was conducted according to the method described by Brand-Williams (14 Brand-Williams *et al.*, 1995) and carried out on 96 wells microplate. The percentage of DPPH remaining was calculated as a function of the molar ratio of antioxidant to DPPH: 

DPPH% = ((DO_control_ - DO_sample_)/DO_control_) × 100 

The EC_50_ value (mol/L antiox)/(mol/L DPPH), defined as the effective concentration of antioxidant necessary to decrease the initial DPPH concentration by 50% (14), was calculated from the results by linear regression analysis. The antiradical power (ARP) was calculated as 1/EC_50_: the highest ARP is associated with the greatest DPPH scavenging effect. Evaluation of free radical-scavenging activity was performed with Trolox equivalent antioxidant capacity (TEAC). TEAC value is based on the ability of the antioxidant to scavenge the radical DPPH and is defined as 

TEAC = ARP_(compound) _/ARP_(Trolox)_

*Measurement of the ABTS-scavenging activity*

For the ABTS assay, the procedure followed the method of Arts *et al.* (15, 16) with some modifications. This test was carried out on 96 wells microplates and compounds were dissolved in MeOH. The absorbance was measured at 734 nm using the spectrophotometer. The remaining percentage of ABTS^.+^ was calculated as a function of the molar ratio of antioxidant to ABTS^.+^ using the following Equation: 

ABTS^.+^% = ((DO_control_ - DO_sample_)/DO_control_) × 100 

The evaluation of the radical-scavenging activity in this method is identical to DPPH method (EC_50_, TEAC, ARP).

*Antioxidant capacity by CUPRAC*

The test CUPRAC (Cupric ion reducing Antioxidant Capacity) was first described by Apak *et al.*, (17). It is based on the capacity to use the copper (II)-neocuproine reagent as the chromogenic oxidizing agent. This test was carried out on 96 wells microplates. The absorbance was measured at 450 nm using the spectrophotometer.

The antioxidant capacity of the compounds and extracts were evaluated as Trolox equivalents (TEAC values). If the results are exposed as DO = f (concentration of compound in g/L), TEAC could be calculated as E‰_(compound)_/E‰_(Trolox)_ where E‰ represents the slope for each curve obtained.

## Results and Discussion

**Figure 1.**
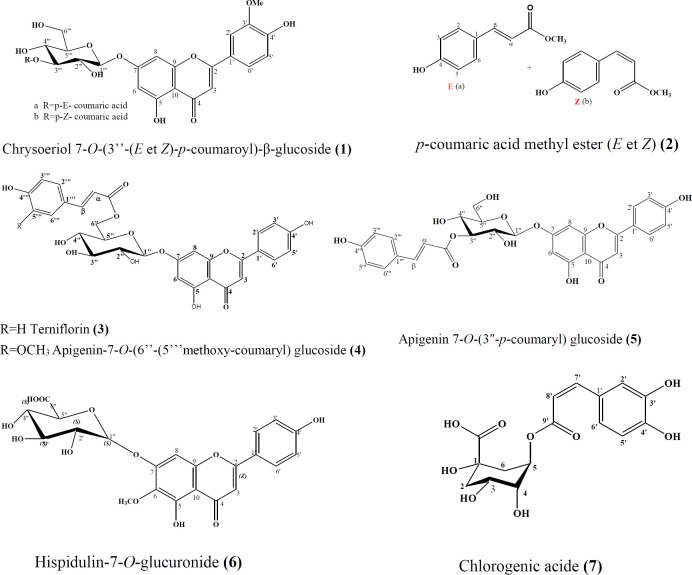
Chemical structures of compounds **1–7** isolated from *Phlomis bovei* de Noé

The methanolic extract of the aerial parts of *Phlomis* *bovei* was fractionated and purified by combination of chromatographic methods to obtain five known flavones glucosides, and two cinnamic acid derivatives. Chrysoériol 7-*O*-(3’’-*p*-coumaroyl)-β-glucoside **(1)** (18), and *p*-coumaric acid methyl ester **(2) **(19), were identified as mixture of *E* and *Z* forms, beside terniflorin (apigenin-7-*O*-(6’’-*E-p*-coumaroyl) glucoside) **(3)** (20), apigenin-7-*O*-(6’’-(5’’’methoxy-coumaryl) glucoside **(4) **(21), apigenin 7-*O*-(3″-*p*-coumaryl) glucoside **(5)** (22)*, *hispidulin-7-*O*-glucuronide **(6)** (23), and chlorogenic acid **(7) **(24) (Figure 1).

Compound **1a**, Chrysoeriol 7-*O*-(3’’-(*E)*-*p*-coumaroyl)-β-glucoside: yellow solid

^1^HNMR (400 MHz, C_3_D_6_O). Aglycon moiety: d 6.70 (1H, s, H-3), d 6.45 (1H, m, H-6), d 6.84 (1H, d, *J* = 6.84 Hz, H-8), d 7.55 (1H, m, H-2’), d 6.93 (1H, d, *J* = 8.8 Hz, H-5’), d 7.90 (1H, d, *J* = 7.85 Hz, H-6’), δ 3.94 (3H, S, OMe), Glucose moiety: δ 5.22 (1H,m, H-1’’), δ 5.18 (1H, m, H-3’’), δ 3.92-3.63 (1H, signal patterns unclear due to overlapping, H2’’, H4’’, H5’’, H6’’), coumaroyl moiety: d 6.39 (1H, d, *J =* 15.7 Hz, H-a), d 7.63 (1H, d, *J* = 15.7 Hz, H-β), d 7.71 (2H, d, *J* = 8.5 Hz, H-2’’’/6’’’), d 6.81 (1H, d, *J* = 8.5 Hz, H3’’’/5’’’),. ^13^C NMR (100 MHz, C_3_D_6_O) d 164.3 (C2), d 102.7 (C3), d 177.6 (C4), d 156.2 (C5), d 99.1 (C6), d 165.4 (C7), d 94.1 (C8), d 150.2 (C9), d 101.6 (C10), d 121.4 (C1’), d 119.9 (C2’), d 147.4 (C3’), d 160.6 (C4’), d 114.8 (C5’), d 127.7 (C6’), d 54.8 (OMe), d 99.5 (C1’’), d 71.1 (C2’’), d 76.6 (C3’’), d 67.5 (C4’’), d 76.3 (C5’’), d 60.3 (C6’’), d 113.9 (Ca), d 144.2 (Cβ), d 125.1 (C1’’’), d 129.2 (C2’’’/6’’’), d 114.9 (C3’’’/5’’’), d 159.2 (C4’’’), d 166.2 (COO). UV spectrum, bands II and I respectively (MeOH, λmax, nm): 269, 316. 

 Compound **1b**, Chrysoeriol 7-*O*-(3’’-(*Z)*-*p*-coumaroyl)-β-glucoside: ^1^HNMR and ^13^CNMR (400 MHz, C_3_D_6_O): Glucose moiety: δ 5.18 (1H, m, H-3’’), coumaroyl moiety: d 5.83 (1H, d, *J =* 12.9 Hz, H-a), d 6.86 (1H, d, *J* = 12.9 Hz, H-β), d 7.50(2H, d, *J* = 8.5Hz, H-2’’’/6’’’), δ 6.74 (2H, d, *J* = 8.5 Hz, H3’’’/5’’’),. ^13^C NMR (100 MHz, C_3_D_6_O) d 76.9 (C3’’), d 115.2 (Ca), d 142.4 (Cβ), d 125.6 (C1’’), d 132.0 (C2’’’/6’’’), d 113.8 (C3’’’/5’’’), d 158.0 (C4’’’), d 165.4 (COO).

Figure1, compound **2a**, *p*-coumaric acid methyl ester (E) : amorphous solid ^1^HNMR (400 MHz, CD_3_OD): d 7,36 (1H, d, *J *= 9.0 Hz, H-2), d 6, 68 (2H, d, *J *= 9.0 Hz, H-3/5), d 7,36 (1H, d, *J *= 9.0 Hz, H-6), d 6,19 (1H, d, *J *= 15.0 Hz, H-a), d 7,58 (1H, d, *J *= 15.0 Hz, H-β), d 3,77 (3H, s, Me). ^13^C NMR (100 MHz, CD_3_OD): d 124.9 (C1), d 131.3 (C2/6), d 118.3 (C3/ 5), d 166.0 (C4), d 112.8 (Ca), d 147.4 (Cβ), d 170.4 (COO), d 51.6 (Me).

Compound **2b**, *p*-coumaric acid methyl ester (Z) : ^1^HNMR (400 MHz, C_3_D_6_O): d 7,6 (1H, d, *J *= 9.0 Hz, H-2/6), d 6,81 (1H, d, *J *= 9.0 Hz, H-3), d 5,67 (1H, d, *J *= 15 Hz, H-a), d 3,71(3H, s, Me). ^13^C NMR (100 MHz, CD_3_OD): d 126.6 (C1), d 133.9 (C2/6), d 116.7 (C3/5), d 162.8 (C4), d 114.9 (Ca), d 145.6 (Cβ), d 169.0 (COO), d 51.4 (Me).

 Compound **3, **Terniflorin (apigenin-7-*O*-(6’’-*E-p*-coumaroyl)glucoside: white powder, ^1^HNMR (400 MHz, DMSO-d6): Aglycon moiety: d 6.79 (1H, s, H-3), d 6.46 (1H, d, *J *= 2.0 Hz, H-6), d 6.79 (1H, d, *J *= 2.0 Hz, H-8), d 7.91 (2H, d, *J *= 8.7 Hz, H-2’/6’), d 6.90 (2H, d, *J *= 8.8 Hz, H-3’/5’), d 12.97 (1H, s, OH-5), Glucose moiety: δ 5.16 (1H, d, *J* = 7.0 Hz, H-1’’), δ 5.18 (1H, m, H-3’’), δ 4.16-3.25 (1H, signal patterns unclear due to overlapping, H2’’, H3’’, H4’’, H5’’, H6’’), coumaroyl moiety: d 6.31 (1H, d, *J =* 16.0 Hz, H-a),d 7.48 (1H, d, *J* = 16.0 Hz, H-β), d 7.35(2H, d, *J* = 8.5Hz, H-2’’’/6’’’), d 6.67 (2H, d, *J *= 8.5 Hz, H3’’’/5’’’). ^13^C NMR (100 MHz, DMSO-d6): d 162.9.6 (C2), d 102.8 (C3), d 182.2 (C4), d 161.3 (C5), d 99.4 (C6), d 164.4 (C7), d 94.7 (C8), d 150.8 (C9), d 105.4 (C10), d 120.7 (C1’), d 128.7 (C2’/6’), d 116 (C3’/5’), d 161.6 (C4’), d 99.4 (C1’’), d 73.0 (C2’’), d 76.1 (C3’’), d 69.9 (C4’’), d 74.0 (C5’’), d 63.6 (C6’’), d 113.8 (Ca), d 145.0 (Cβ), d 124.5 (C1’’’), d 130.2 (C2’’’/6’’’), d 115.7 (C3’’’/5’’’), d 160.0 (C4’’’), d 166.5 (CO). UV spectrum bands II and I respectively (MeOH, λmax, nm): 269, 320. Which was further confirmed by a positive HR-ESI-MS analysis (m/z 577[M-H]^-^).

Compound **4, **Apigenin-7-*O*-(6’’-(5’’’methoxy-coumaroyl)) glucoside: white powder, ^1^HNMR (400 MHz, DMSO-d6): Aglycon moiety: d 6.68 (1H, s, H-3), d 6.49 (1H, d, *J *= 2.0 Hz, H-6), d 6.79 (1H, d, *J *= 2.0 Hz, H-8), d 7.89 (1H, d, *J *= 8.7 Hz, H-2’), d 6.96 (1H, d, *J *= 8.8 Hz, H-3’), d 6.84 (1H, d, *J *= 8.8 Hz, H-5’, d 7.49 (1H, d, *J *= 8.8 Hz, H-6’), Glucose moiety : d 5.16 (1H, d, *J *= 7.0 Hz, H-1’’), d 3.54 (1H, m, H-2’’), d 3.60 (1H, m, H-3’’), d 3.44 (1H, m, H-4’’), d 3.90 (1H, m *J *= 2.0, Hz, H-5’’), d 4.59-4.22 (2H, m, H-6’’), Coumaroyl: d 6.26 (1H, d, *J *= 16 Hz, H-a), d 7.47 (1H, d, *J* = 16 Hz, H-β), d 7.55 (1H, s, H-2’’’), d 7.52 (1H, s, H-6’’’), d 3.89 (3H, s, H-8), ^13^C NMR (100 MHz, CMSO-d6) : d 162.4 (C2), d 100.7 (C3), d 180.0 (C4), d 154.9 (C5), d 97.3 (C6), d 164.7 (C7), d 92.6 (C8), d 158.8 (C9), d 103.3 (C10), d 122.9 (C1’), d 126.1 (C2’), d 116.3 (C3’), d 163.2 (C4’), d 113.4 (C5’), d 127.7 (C6’), d 97.4 (C1’’), d 70.6 (C2’’), d 73.9 (C3’’), d 68.0 (C4’’), d 71.6 (C5’’), d 61.3 (C6’’), d 111.3 (Ca), d 143.0 (Cβ), d 124.2 (C1’’’), d 107.3 (C2’’’), d 145.7 (C3’’’), d 148.6 (C4’’’), d 107.3(C5’’’), d 118.2 (C6’’’), d180.0 (CO), d 53.3 (OMe). Which was further confirmed by a positive HR-ESI-MS analysis (m/z 607[M-H]^-^).

 Compound **5**, Apigenin-7-O-(3’’-*p*-coumaroyl) glucopyranoside: yellow powder. ^1^HNMR (400 MHz, CD_3_COCD_3_+D_2_O): Aglycon moiety: d 6.69 (1H, s, H-3), d 6.47 (1H, s, H-6), d 6.83 (1H, d, *J *= 7.8 Hz, H-8), d 7. 91 (2H, d,* J* = 7.8 Hz, H-2’/6’), d 6. 98 (2H, d, *J = *7.5 Hz, H-3’/5’), Glucose moiety: d 5.28 (1H, d, *J = *6.3 Hz, H-1’’), d 3.70 (1H, t, *J = *8.0 Hz, H-2’’), d 5.18 (1H, t, *J = *8.0 Hz, H-3’’), d 3.67 (1H, m, H-4’’), d 3.75 (1H, m, H-5’’), d 3.89-3.74 (2H, m, H-6’’), coumaroyl moiety: d 6.37 (1H, d, *J = *15.8 Hz, H-a), d 7.62 (1H, d, *J = *15.8 Hz, H-β), d 7.51 (2H, d, *J = *8.0 Hz, H-2’’’/6’’’), d 6.83 (2H, d, *J = *7.8 Hz, H-3’’’/5’’’), ^13^C NMR (100 MHz, CD_3_COCD_3_+D_2_O) : d 165.5 (C2), d 103.2 (C3), d 182.8 (C4), d 161.8 (C5), d 100.2 (C6), d 163.4 (C7), d 95.4 (C8), d 157.5 (C9), d 106.0 (C10), d 121.7 (C1’), d 128.9 (C2’/6’), d 116.3 (C3’/5’), d 163.4 (C4’), d 100.3 (C1’’), d 71.8 (C2’’), d 77.4 (C3’’), d 68.3 (C4’’), d 77.0 (C5’’), d 61.1 (C6’’), d 114.6 (Ca), d 145.6 (Cβ), d 126.0 (C1’’’), d 130.4 (C2’’’/6’’’), d 116.1 (C3’’’/5’’’), d 160.0 (C4’’’), d 167.6 (COO). UV spectrum bands II and I respectively (MeOH, λmax, nm): 270, 317. Which was further confirmed by a negative FAB-MS analysis (m/z 577 [M-H]^-^).

Compound **6**, Hispidulin-7-*O*-glucuronide: yellow powder. ^1^HNMR (400 MHz, CD_3_OD): Aglycon moiety: d 6.65 (1H, s, H-3), d 6.97 (1H, s, H-8), d 7.88 (2H, d, *J = *8.5 Hz, H-2’/6’), d 6.92 (1H, d, *J = *8.8 Hz, H-3’/5’), d 3.89 (3H, s, OMe), Glucuronide moiety: d 5.20 (1H, d, *J = *7.5 Hz, H-1’’’), d 3.61 (1H, m, H-2’’), d 3.59 (1H, m, H-3’’), d 3.62 (1H, m, H-4’’), d 4.03 (1H, d, *J = *9.4 Hz, H-5’’), ^13^C NMR (100 MHz, CD3OD): d 166.9 (C2), d 103.7 (C3), d 184.4 (C4), d 154.1 (C5), d 134.3(C6), d 157.7 (C7), d 95.8 (C8), d 154.1 (C9), d 107.7 (C10), d 123.1 (C1’), d 129.7 (C2’/6’), d 117.0 (C3’/5’), d 162.9 (C4’), d 61.5 (OMe), d 101.8 (C1’’), d 74.5 (C2’’), d 77.5 (C2’’), d 77.5 (C3’’), d 73.1 (C4’’), d 76.6 (C5’’), d 174.0 (C6’’). Which was further confirmed by a negative ESI-MS analysis (m/z 475 [M-H]^-^).

Compound **7, **Chlorogenic acid: ^1^HNMR (400 MHz, CD_3_OD): d 2.16-2.02 (2H, m, H-2), d 4.18 (1H, m, H-3), d 3.72 (1H, m, H-4), d 5.32 (1H, d, *J = *3.8 Hz, H-5), d 2.20-2.06 (2H, m, H-6), d 7.03 (1H, d, *J = *1.9 Hz, H-2’), d 6.77 (1H, d, *J = *8.2 Hz, H-5’), d 6.94 (1H, d, *J = *1.9 Hz, H-6’), d 7.55 (1H, d, *J = *15.75 Hz, H-7’), d 6.29 (1H, d, *J = *16.1 Hz, H-8’), ^13^C NMR (100 MHz, CD3OD): d 76.8 (C1), d 38.4 (C2), d 71.9 (C3), d 73.7 (C4), d 72.1 (C5), d 39.3 (C6), d 127.8 (C1’), d 115.2 (C2’), d 146.8 (C3’), d 149.6 (C4’), d 116.5 (C5’), d 123.0 (C6’), d 147.0 (C7’), d 115.3 (C8’), d 168.8 (C9’), d 173.8 (COO). Which was further confirmed by a negative ESI-MS analysis (m/z 353 [M+H]^+^).

 The different extracts from *P. bovei* were tested for the ability to scavenge DPPH and ABTS^+^ free radicals and also for the capacity to reduce the cupric ion. The results are presented in Table 1. The methanolic and hydromethanolic extracts showed the most significant antiradical activities towards the 1,1-diphenyl 2-picrylhydrazyl (DPPH) whereas the methanolic extract had a good antioxidant activity measured with the CUPRAC test with a TEAC = 0.23.

The other extracts exhibited a low antiradical activity since TEACs are less than 0.2 with DPPH, ABTS, or CUPRAC.

The activities of the phenolic compounds **1- 5** isolated from the MeOH extract were measured *via* the DPPH, ABTS, and CUPRAC tests at different concentrations taking trolox as the positive drug (Table 2). 

The five compounds isolated showed no measurable radical scavenging activity toward DPPH. 

The comparison between the antioxidant activity of compounds **3** and **5** shows that the TEAC measured with the CUPRAC test reveals large disparities. Compound **3** is almost 4 times more active than trolox (TEAC_CUPRAC _= 3.68 *vs.* TEAC_CUPRAC _= 1.00 for Trolox), while compound **5** has an insignificant activity (TEAC = 0.04). These results are consistent in a sense with those described by Apak *et al.* (17 2004), which attribute a strong effect to the presence of a strong conjugated structure as in the cinnamic acid derivatives (Ar-CH = CH-COO), which cannot then be attacked by the cupric ion. However, our results show that this can be nuanced depending on the position of the coumaryl group on sugar. The presence of Z/E isomers is favorable to reduce the antioxidant capacity measured with CUPRAC as shown with TEAC values of compound **1** or **2** (TEAC = 0.14 and TEAC = 0.03 respectively). It is the same for the substitution by a methoxy group in *meta* position of the para-coumaryl nucleus (compound **4**).

The TEAC value of compounds **1 **(TEAC = 0.13) and **4** (TEAC = 0.19) were more active than the reference (rutin) (TEAC = 0.11) concerning radical scavenging properties toward ABTS^+^.

Concerning the genotoxicity studies, compound **3** was evaluated as non-genotoxic at all tested concentrations (Table 3). Whereas compounds **1** (at 10 µg/assay), and **4** (at 50 µg/assay) are shown to be marginally genotoxic at the indicated concentrations. Therefore, DNA is not considered as a target for these compounds.

On the other hand, we studied the protective effect of flavones **1, 3,** and **4** on NF induced damage. Dose of 10 µg/assay of NF was chosen for the antigenotoxicity studies, since this dose was not toxic and induced a significant SOS response. It is the dose that gives the maximum of genotoxicity for NF. As shown in Table 4, compounds **1** and **3** reduce the genotoxicity induced by NF moderately by 86.6% and 64.6%, respectively, at the highest dose of 50 μg/assay. Lastly, compound **4** was the one showing the strongest activity, since it remains close to 90% of genotoxicity inhibition at low dose (2 μg/assay). 

Flavonoids **3** and **4** are shown to possess a considerable antimutagenic potency in our experiments. This result can be explained by the presence of a pyrone group (nucleus C), two hydroxyl groups on C-4/C-5 in the flavonoid structure, as reported previously by Krizkova *et al.* (25) and Edenharder *et al.* (26). On the contrary, compound **1** showed the lowest diminution of antimutagenic effect compared to compounds **3** and **4**, this effect could probably be ascribed to the methylation of the 3’-hydroxyl function as hypothesized by Edenharder *et al.* (26).

**Table 1.** Antioxidant activities of Extracts from *Phlomis bovei *de Noé

**Extrait**	**ABTS**			**CUPRAC**		**DPPH**		
	IC_50_	ARP	TEAC	E‰	TEAC	IC_50_	ARP	TEAC
DCM	3,08	0.32	0.01	0.01	0.04	>476	-	-
H_2_O	0.40	2.5	0.1	0.04	0.19	1.49	0.67	0.08
MeOH	0.23	4.34	0.17	0.03	0.14	0.26	3.85	0.46
H_2_O+MeOH	0.23	4.34	0.17	0.05	0.23	0.24	4.17	0.50

**Table 2:** Antioxidant activities of phenolic compounds from *Phlomis* *bovei* de Noé.

	**ABTS**			**CUPRAC**		**DPPH**			
	**IC**_50_^*^	**ARP**	**TEAC**	**E‰ (L/g/cm)**	**TEAC**	**IC**_50_^**^	**ARP**	**TEAC**
Trolox	0.16	6.1	1.0	0.22	1.00	0.42	2.4	1.0
Rutine	0.34	2.9	0.11	0.66	3.3			
								
1	0.29	3.4	0.13	0.03	0.14	>0.5	-	-
2	0.99	1.0	0.04	0.007	0.03	>0.5	-	-
3	0.79	1.3	0.08	0.79	3.68	Oxydant ou Prooxydant^a^	-	-
5	0.52	1.9	0.07	0.01	0.04	>0.5	-	-
4	0.21	4.8	0.19	0.13	0.05	>0.5		

^a^Percentage of DPPH.remaining higher than 100%.

^*^In (mol/lantiox)/(mol/lABTS_+).

^**^In (mol/lantiox)/(mol/lDPPH_).

**Table 3.** Genotoxic activity of *Phlomis bovei* de Noé compounds by the SOS Chromotest in the presence of *Escherichia coli* PQ37

**Compounds**	**Doses (μg/assay)**	**β-gal (U)**	**AP (U)**	**R**	**IF**
**NC**		3.00 ± 0.02	21.40 ± 0	0.14	
**NF**		70.51 ± 0	15.32 ± 0	4.60	32.8
**1**	50 10 2	2.81 ± 0.02 4.43 ± 0.01 1.77 ± 0.02	16.88 ± 0.01 18.38 ± 0.01 14.52 ± 0.04	0.16 0.24 0.12	1.1 1.7 0.8
**3**	50 10 2	2.09 ± 0 2.28 ± 0.05 2.36 ± 0.03	15.55 ± 0 15.16 ± 0.01 15.33 ± 0.01	0.13 0.15 0.15	1.0 1.1 1.1
**4**	50 10 2	4.03 ± 0.03 3.10 ± 0.02 3.03 ± 0	14.83 ± 0 17.55 ± 0.03 15.55 ± 0.04	0.27 0.17 0.19	1.9 1.2 1.4

β-Gal: β-galactosidase; AP: alkaline phosphatase; U: enzyme units; IF: induction factor; NC: negative control (nontreated cells); NF: nitrofurantoin, positive control of genotoxicity.

**Table 4:** Effect of *Phlomis bovei* de Noé compounds on the genotoxicity induced by NF (10  g/assay) in the presence of *Escherichia coli* PQ37

**Composés**	**Doses (μg/essai)**	**β-gal (U)**	**AP (U)**	**IF**	**IP (%)**
NC		3.40 ± 0.01	17.9 ± 0.05		
NF		15.35 ± 0.04	5.4 ± 0.06	14.95	
1	50 10 2	9.10 ± 0.01 10.53 ± 0 11.00 ± 0	16.71 ± 0 16.02 ± 0 16.50 ± 0.01	2.86 3.45 3.50	86.6 82.4 82.0
3	50 10 2	20.31 ± 0.02 19.44 ± 0 20.10 ± 0.02	17.9 ± 0 16.94 ± 0 16.58 ± 0.02	5.93 6.02 6.37	64.6 63.9 61.5
4	50 10 2	4.70 ± 0 6.69 ± 0 8.45 ± 0	17.40 ± 0.02 16.20 ± 0.01 16.00 ± 0.01	1.42 2.17 2.77	96.9 91.5 87.2

β-Gal: β-galactosidase; AP: alkaline phosphatase; U: enzyme units; IF: induction factor; IP: inhibition percentage; NC: negative

control (nontreated cells); NF: nitrofurantoin,positive control of genotoxicity.

## Conclusion

Five known flavonoids and two cinnamic acid derivatives, were isolated from the aerial parts of *Phlomis bovei *de Noé used for its medicinal properties. Compound **4** has been described for the first time inthe genus *Phlomis* and in the Lamiaceae family.

The antiradical activity of the polar extracts depends on the type of radical to be trapped. But these extracts have a chelating effect, hence an antioxidant activity.

On the other hand, non polar extracts have no antioxidant effect (antiradical/chelation of metals).

For compounds isolated from *Phlomis bovei*, it has been noted that most flavonoid compounds do not exhibit significant antioxidant activity except compound **3** which showed a high activity concerning radical scavenging properties toward ABTS+ compared to trolox.

As a correlation between antigenotoxic and antiradical activities has been established by Park *et al.* (27), this led us to test the isolated products. The results obtained show the interest of compound **3** to inhibit the genotoxicity of nitrofurantoin but the compound **4** exhibits antigenotoxicity at different concentrations.
